# Retraction: Correlations between parameters of glycaemic variability and foetal growth, neonatal hypoglycaemia and hyperbilirubinemia in women with gestational diabetes

**DOI:** 10.1371/journal.pone.0338857

**Published:** 2025-12-18

**Authors:** The PLOS One Editors

The *PLOS One* Editors retract this article [1] because it was identified as one of a series of submissions for which we have concerns about compromised peer review.

All authors either did not respond directly or could not be reached.
